# Early Rising

**Published:** 1876-08

**Authors:** 


### *EARLY RISING.*

The habits of an industrious and thrifty people should and do conform to their necessities. "Early to bed and early to rise," was a beautiful and encouraging sentiment during the early years of the Republic, when men were subduing the wilderness unaided by that mighty power which is now attached to every industry. We have shifted our heaviest burdens to the shoulders of this submissive giant, and may now enjoy our "*otium cum dignitate*," in a land "flowing with milk and honey." Still we are not realizing the miseries predicted by the gloomy croakers of the past generation. We are not the effeminate and short-lived people they predicted we should be, but a hale, stalwart race whose chances of life and old age are double what theirs were. *Our modesty* forbids us to refer to the influence that HALL'S JOURNAL OF HEALTH has exerted through its wise counsels, in attaining results so satisfactory. It has certainly maintained that wear and hard work does not contribute to the preservation of health and life, any more than the rust of indolence. It has taught that industry—not drudgery—is essential to health and happiness.

Sufficient sleep, rest, and food are necessary to health and to prosperity in all the affairs of life. A human body weakened and irritated by want of sleep and sufficient food, is just as unfitted for efficient labor as a crippled horse is for a race course.

If we always treated ourselves with the same care that self interest inspires in the care and treatment of our domestic animals, we should escape many ills, as they do.

